# Core Outcomes in Ventilation Trials (COVenT): protocol for a core outcome set using a Delphi survey with a nested randomised trial and observational cohort study

**DOI:** 10.1186/s13063-015-0905-9

**Published:** 2015-08-20

**Authors:** Bronagh Blackwood, Suzanne Ringrow, Mike Clarke, John Marshall, Louise Rose, Paula Williamson, Danny McAuley

**Affiliations:** Centre for Infection and Immunity, Queen’s University Belfast, Health Sciences Building, 97 Lisburn Road, Belfast, BT9 7AE Northern Ireland; Northern Ireland Network for Trials Methodology Research, Centre for Public Health, Queen’s University Belfast, Institute of Clinical Sciences, Block B, Royal Victoria Hospital, Belfast, BT12 6BA Northern Ireland; Li Ka Shing Knowledge Institute, St Michael’s Hospital, Toronto, ON Canada; Sunnybrook Health Sciences Centre, Toronto, ON Canada; Lawrence S Bloomberg Faculty of Nursing, University of Toronto, Toronto, ON Canada; Provincial Centre of Weaning Excellence, Toronto East General Hospital, Toronto, ON Canada; West Park Healthcare Centre, Toronto, ON Canada; Department of Biostatistics, University of Liverpool, Shelley’s Cottage, Brownlow Street, Liverpool, L69 3GS UK; Regional Intensive Care Unit, Royal Victoria Hospital, Belfast, BT12 6BA Northern Ireland

**Keywords:** Consensus methods, Core outcome set, Critical care, Delphi technique, Mechanical ventilation

## Abstract

**Background:**

Among clinical trials of interventions that aim to modify time spent on mechanical ventilation for critically ill patients there is considerable inconsistency in chosen outcomes and how they are measured. The Core Outcomes in Ventilation Trials (COVenT) study aims to develop a set of core outcomes for use in future ventilation trials in mechanically ventilated adults and children.

**Methods/design:**

We will use a mixed methods approach that incorporates a randomised trial nested within a Delphi study and a consensus meeting. Additionally, we will conduct an observational cohort study to evaluate uptake of the core outcome set in published studies at 5 and 10 years following core outcome set publication. The three-round online Delphi study will use a list of outcomes that have been reported previously in a review of ventilation trials. The Delphi panel will include a range of stakeholder groups including patient support groups. The panel will be randomised to one of three feedback methods to assess the impact of the feedback mechanism on subsequent ranking of outcomes. A final consensus meeting will be held with stakeholder representatives to review outcomes.

**Discussion:**

The COVenT study aims to develop a core outcome set for ventilation trials in critical care, explore the best Delphi feedback mechanism for achieving consensus and determine if participation increases use of the core outcome set in the long term.

## Background

The provision of mechanical ventilatory support with accompanying sedation is a major supportive therapy in intensive care units (ICUs), but is not without potential harms. In an attempt to minimise these, research has examined the effect of various interventions to reduce unnecessary time receiving mechanical ventilation. These interventions include sedation and ventilator weaning protocols, and alternative ventilation modes. Systematic reviews of trials spanning 20 years that evaluated protocolised and automated weaning in adults and children highlight variation in how outcomes were defined, measured and reported [1–3]. Outcome variability is not limited to trials of protocolised weaning. A systematic review of 66 critical care trials that measured ventilation as a primary or secondary outcome found outcome reporting was not standardised [4]. Of 48 trials reporting duration of mechanical ventilation, 75 % did not define start and end points, and the remaining 25 % used variable definitions. Furthermore, of 25 trials measuring ventilator-free days, 36 % did not provide a definition, that is, when counting of ventilator-free days commenced and ended; of those that did, again variability was noted. Notably, although all 66 trials measured ventilation outcomes, no two trials used the same ‘set’ of outcomes to do this.

It is evident there is a need for standardisation of outcome selection and definitions, and difficulties raised by inconsistent outcome reporting are increasingly recognised. The Consolidated Standards of Reporting Trials (CONSORT) statement 2010 [5] and the Standard Protocol Items: Recommendations for Interventional Trials (SPIRIT) statement 2013 [6] both explicitly advocate the use of ‘core outcome sets’ to achieve consistency in outcome reporting. These statements refer researchers to the Core Outcome Measures in Effectiveness Trials (COMET) database for guidance [7]. A core outcome set (COS) is defined as ‘the minimum [number of outcomes] that should be measured and reported in all clinical trials of a specific condition’ [8]. The COMET Initiative was commissioned to encourage the development and application of COS across health and social care, create accessible information about guidelines on outcome selection, and facilitate collaboration among researchers developing COS. The initiative has received widespread international support from organisations, including Outcome Measures in Rheumatology (OMERACT) [9] and the International Forum for Acute Care Trialists (InFACT) [10]. InFACT aims to promote international collaboration in critical care research and address barriers in undertaking trials, and is a major driving force behind the advancement of creating COS for critical care trials [10].

Using a Delphi study followed by a consensus meeting is a widely accepted method of achieving consensus for developing COS [8], but there are variations in how feedback to the panel is provided during Delphi rounds. Currently, there is no evidence as to which feedback method will best achieve consensus that is representative of all stakeholder groups. A systematic review of studies using the Delphi technique to develop COS found no evidence to identify which methods will yield consensus that is genuinely representative of all stakeholder groups [11]. When developing a COS important factors to take into consideration are the inclusiveness of stakeholders in the Delphi panel and the mechanism used to provide feedback to the panel. Furthermore, once developed, it is important to evaluate subsequent COS uptake in clinical trials. Our Delphi study to develop a COS will address both variation in selecting and defining outcome measures for ventilation trials, and will lay the foundation for follow-up to examine uptake.

### Aims and research questions

The primary aim is to develop a COS for trials involving patients undergoing mechanical ventilation where an aim of the intervention is to modify time spent on mechanical ventilation. The secondary aims are to determine the most valid method of gaining consensus for a COS, and to evaluate the COS uptake 5 and 10 years after its publication.

Our research questions are:

1. Which outcomes are ranked by stakeholders as critical for inclusion in the COS?
2. How is the ranking process influenced by the method of stakeholder group feedback during Delphi rounds?
3. Do Delphi panel participants rank the importance of outcomes differently depending on their knowledge of the other groups’ ranking?
4. Does participation by investigators in the Delphi panel affect their subsequent use of the COS, compared with investigators who do not participate?

## Methods

We will use a mixed methods approach that incorporates a randomised trial nested within a Delphi study and a consensus meeting. Subsequently, we will conduct an observational cohort study to evaluate COS uptake in published studies at 5 and 10 years following COS publication.

### Delphi study

We will use the Delphi technique to achieve input from a broadly representative international and diverse stakeholder panel. Our Delphi study consists of a series of sequential questionnaires or ‘rounds’ aiming to obtain a consensus of opinion from a group of expert stakeholders [12]. The Delphi technique can also minimise response bias as individual feedback is anonymised and not influenced by views of influential individuals.

### The Delphi panel

A range of expertise within the panel is considered to be an important quality criterion for development of COS [11]. We will therefore seek to include representatives from seven key stakeholder groups that may be particularly interested in this COS. The groups and inclusion criteria are as follows:

1. Industry and pharmaceutical representatives – from companies producing technology and products used in mechanical ventilation.
2. Nurses and allied health professionals (NAHP) – members of professional societies or associations who have a primary role in clinical critical care practice with a minimum of 5 years’ experience.
3. Physicians – members of professional societies or associations who are consultant grade (or equivalent) in intensive care, pulmonology or anaesthetics, and have a primary role in clinical practice.
4. Research funding organisations – representatives from funding bodies that have funded or commissioned critical care research projects in the last 5 years.
5. Patient support groups or charities – representatives from respiratory or critical care charities; patient or relatives user groups, forums or support groups, preferably with personal experience of critical care.
6. Clinical trial groups (CTGs) – members of CTGs that may include the Chair and other appropriate members, preferably representing diverse professions.
7. Trial investigators – primary and senior authors of reports of clinical trials evaluating interventions aiming to reduce duration of mechanical ventilation. These will be identified from a systematic search of publications in PubMed over the last 10 years.

### Panel size and recruitment

There are no generally accepted guidelines for optimal panel size to achieve stable consensus in Delphi studies [13]. Panel size in other projects has usually been guided by practicality, question scope and time available for analysis [11]. We will attempt to recruit as large a panel as possible, and will encourage all organisations within each stakeholder group to nominate between five and ten representatives who meet the inclusion criteria.

Organisations within each stakeholder group will be identified through our wide network of critical care contacts and through a methodological web-based search. We will email study information to key contacts (for example, Chair, President, Director) within organisations, inviting them to nominate expert participants or to forward a recruitment letter.

The French Cochrane Centre will undertake a systematic search of PubMed to identify trial investigators. This search will include appropriate terms for: population (adult and child); interventions (aimed at reducing duration of mechanical ventilation); year of publication (2004–2014); and study type (randomised trials, observational studies). We anticipate an overlap between eligible participants identified through the PubMed search and those identified by CTGs; therefore, we will only recruit those participants not identified by the CTGs into the trial investigator arm.

Previous studies of the Delphi technique demonstrate high attrition after four rounds [10]. Therefore, we will use three rounds and provide two-weekly reminders to participants. Other techniques to minimise attrition include: provision of a personalised invitation and reminders; an outline of timelines; and ensuring that each round is concise and easy to complete with minimal time commitment. Participants will receive an email comprising a clear study explanation emphasising the importance of completing all rounds, an estimate of the amount of time to complete the questionnaires and requesting consent for participation.

### The Delphi questionnaire and rounds

The Delphi will be managed using a bespoke online e-management system maintained by the COMET Initiative. This system has previously been used in the Management of Otitis Media with Effusion in Cleft Palate (MOMENT) study developing a COS for trials investigating otitis media with effusion in children [14]. It has been modified for this project to accommodate data collection, randomisation process, web link to the relevant questionnaire and presentation of scoring within each round.

### Round 1

**Fig. 1:**
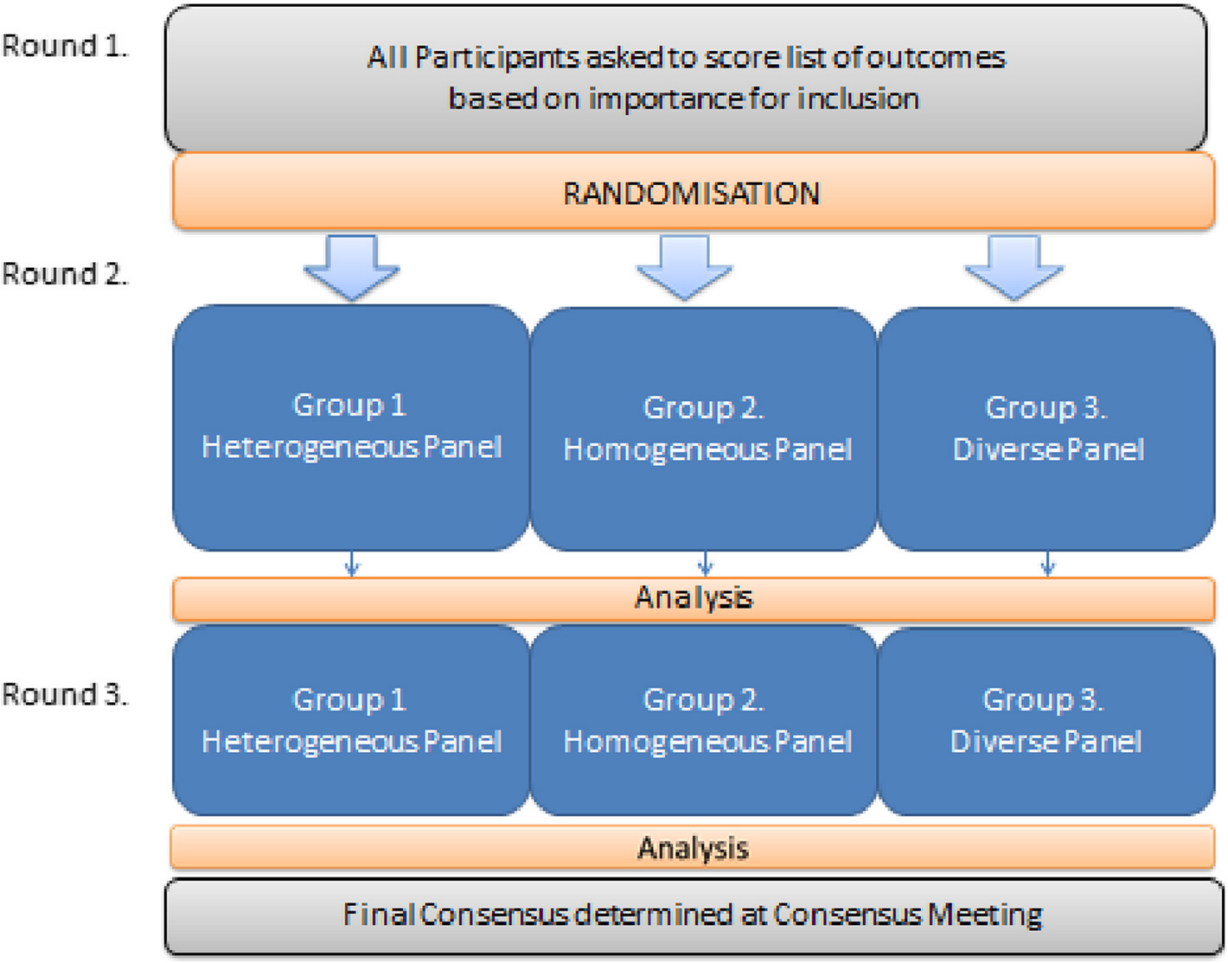
Flowchart of the Delphi study and nested randomised controlled trial

We will use the most frequently reported outcomes identified from our systematic review for the first Delphi round. Participants will be asked to score each outcome using the Grading of Recommendations Assessment, Development and Evaluations (GRADE) scale of 1–9, where 1 represents least important and 9 represents most important [15]. Participants will have the option to suggest additional outcomes for inclusion in the second round. We will list outcomes in alphabetical order to avoid potential weighting. Outcomes suggested during round 1 will be reviewed and coded by two research team members to ensure they represent new outcomes; where uncertainty exists, the advisory group will be consulted. Scores attributed to each outcome will be calculated as a percentage of the total responses for all scores. This will be summarised by stakeholder groups and all outcomes will be carried forward to round 2. A flowchart of the study is shown in Fig. 1.

We will collect demographic information from participants that will be stored in a separate database and used to provide the participant with a unique identifier. Participants will be asked to provide information on their age, background, years of experience, field of interest, working country and current position, to help to define the composition of the panel.

### Randomisation

After round 1, participants will be randomised to one of three groups that will determine the method of feedback they will receive. Participants will be stratified by type of stakeholder to ensure equal representation in each study group. The randomisation process will be conducted through the COMET e-management system.

Study groups are as follows: group 1 will be shown collated responses of the entire panel for each outcome (heterogeneous group); group 2 will be shown collated responses for each outcome only from the stakeholder group to which they belong (homogeneous group); and group 3 will be shown collated responses for each outcome for each stakeholder group (diverse group).

### Rounds 2 and 3

In rounds 2 and 3, all three study groups will receive the same list of outcomes with feedback tailored in accordance with their randomisation. Participants will be asked to reflect on their own response and the collated responses, and score the outcomes again.

### Analysis

During rounds, scores (1–9) will be calculated as a percentage of the total responses. The final responses will be analysed for each of the three study groups. We will define consensus for outcome inclusion in the COS as >70% of responses rating the outcome at 7 or greater and not more than 15% of responses rating the outcome <3 [14]. We will explore similarities and differences across the three COS generated by the different feedback methods. As there is no gold standard for feedback, all three COS will be brought forward to the consensus meeting for consideration and discussion, and for the preparation of a final COS.

### Ethical requirements

Individual participants will be approached (or will self-volunteer) after nomination by an organisational lead from an organisation with which they are affiliated. Participants will be sent a short introductory email with an information sheet about the study, including details of randomisation, explicit details about what will be involved and asked for their consent to participate. Participants will be given the option to withdraw from the study at any time. This study will be conducted entirely online and consistent with standard practice in survey research; consent will be assumed by agreement to participate and completion of online questionnaires. The study was reviewed and approved by the School of Medicine, Dentistry and Biomedical Science Research Ethics Committee at Queen’s University Belfast (QUB), Northern Ireland, UK, in October 2014 [Ref: 14.34v2] (Ethics Committee Reference). Sponsorship and indemnity is provided by QUB.

### Consensus meeting

Following completion of the Delphi, we will invite representatives of stakeholder groups from the Delphi panel, COMET Initiative and the InFACT Outcomes Working Group to the consensus meeting. To promote wide dissemination, we also will invite editors from key journals (for example, *American Journal of Respiratory and Critical Care Medicine*, *Critical Care Medicine*, *British Medical Journal*, *The Lancet*, *Intensive Care Medicine*, *Critical Care*) and funders of critical care trials. The purpose of the consensus meeting is threefold, with the common aim of reaching a consensus among those present on the content of a core outcome set for mechanical ventilation. A report from this meeting will be written up and published.

As described in the paper, participants with differing professional backgrounds in the Delphi study will be randomised into one of three groups, each using a different feedback mechanism between rounds. Therefore, the Delphi will produce three separate proposals for the variables to include in the COS. It is likely that there will be overlap between these three sets but they may also differ in regard to the outcome variable types that they contain. However, given that there is no gold standard in Delphi studies for the best feedback mechanism to attain consensus, we cannot be sure that the consensus on a COS reached by any particular group is superior to that reached by another of the groups, using a different feedback mechanism. The research team will review the consistency or variance between the three COS. If there is consistency, then one COS will be brought to the meeting, but if there is variance we will bring this to the attention of the meeting for discussion and agreement on choosing one particular COS. This is the first purpose of the meeting.

The aim of the Delphi study is only to determine ‘what’ outcomes should be measured in mechanical ventilation trials – it is not designed to determine ‘how’ the outcomes should be measured or, specifically, the time point at which the outcome should be determined. So, the second purpose of the consensus meeting is to bring together experts in mechanical ventilation trials alongside representatives of other organisations contributing to the development and/or dissemination of core outcome measure sets, including OMERACT, InFACT and COMET, to discuss ‘how’ the outcome variables in the COS should be measured.

The third purpose of the consensus meeting is to invite editors from prominent critical care journals to contribute, particularly to discussions on dissemination of the core outcome set on mechanical ventilation.

### Observational cohort study

We will conduct a prospective observational cohort study to determine if participation of trial investigators in the Delphi panel increases COS uptake in comparison with investigators who did not participate, on the basis of the use of the COS in published reports of clinical trials in the decade after the COS is published.

### Sample identification

We will conduct a PubMed search using terms relating to mechanical ventilation, synonyms for ‘duration of mechanical ventilation’ and study design, to identify a cohort of primary and senior authors that have published a trial in this area in the past 10 years.

## Methods

**Fig. 2:**
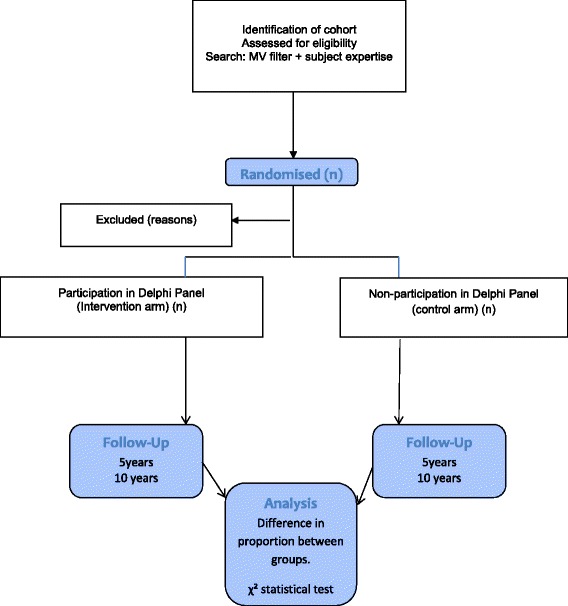
Flowchart of randomisation in the observational cohort study. MV, mechanical ventilation

Study authors will be randomised to either participate in the Delphi panel as a ‘trial investigator’ stakeholder representative or not to participate (see Fig. 2). As we are using data available in the public domain, informed consent will not be sought from those not involved in the Delphi. Participants involved in the Delphi will be asked for informed consent in accordance with other participating stakeholder groups.

### Analysis

The primary outcome is the number and proportion of authors who have used the COS in trials reported at 5 and 10 years after the publication of the COS. Data will be compared between groups using the chi-square test. A *P* value <0.05 will indicate a statistically significant difference.

## Discussion

This paper describes the design of a multi-method study to develop a COS for ventilation trials in critical care, explore the best Delphi feedback mechanism for achieving consensus and determine if participation increases use of the COS in the long term. To our knowledge, it is the first time that the Delphi technique incorporating these methods has been used for developing a COS in this topic area. Other important features of this study are that it will provide guidance for designing Delphi studies for developing COS in critical care and a database of key stakeholder organisations that can be used for development of other critical care COS. In addition to developing the COS, this study may also shed light on controversial aspects within critical care trials.

### Study status

The Delphi study has not yet begun. Delphi panel members are currently being recruited.

## Abbreviations

COMET: Core Outcome Measures in Effectiveness Trials
CONSORT: Consolidated Standards of Reporting Trials
COS: Core outcome set
COVenT: Core Outcomes in Ventilation Trials
CTG: Clinical trial group
GRADE: Grading of Recommendations Assessment, Development and Evaluations
ICU: Intensive care unit
InFACT: International Forum for Acute Care Trialists
MOMENT: Management of Otitis Media with Effusion in Cleft Palate
NAHP: Nurses and allied health professionals
OMERACT: Outcome Measures in Rheumatology
SPIRIT: Standard Protocol Items: Recommendations for Interventional Trials
